# Supplementary material for: Modeling glioblastoma heterogeneity as a dynamic network of cell states
Source: Mol Syst Biol. 2021 Sep 16;17(9):e10105. doi: 10.15252/msb.202010105 (PMC8444284; doi:10.15252/msb.202010105)
Supplement: Supplementary file 6 — Source Data for Figure 5 [file MSB-17-e10105-s004.zip › Figure5A_sourcedata/GSEA_3017/nosigdb_stateA.GseaPreranked.1622795026485/DOROSZKO_U3013MG_CLUSTER_2.html]

Details for gene set DOROSZKO\_U3013MG\_CLUSTER\_2[GSEA]

|  || Dataset | state53017 |
| Phenotype | NoPhenotypeAvailable |
| Upregulated in class | na\_pos |
| GeneSet | DOROSZKO\_U3013MG\_CLUSTER\_2 |
| Enrichment Score (ES) | 0.50227654 |
| Normalized Enrichment Score (NES) | 3.9468744 |
| Nominal p-value | 0.0 |
| FDR q-value | 0.0 |
| FWER p-Value | 0.0 |
Table: GSEA Results Summary

  

Fig 1: Enrichment plot: DOROSZKO\_U3013MG\_CLUSTER\_2      
 Profile of the Running ES Score & Positions of GeneSet Members on the Rank Ordered List

  

| PROBE | GENE SYMBOL | GENE\_TITLE | RANK IN GENE LIST | RANK METRIC SCORE | RUNNING ES | CORE ENRICHMENT || 1 | CDK1 |  |  | 3 | 1.006 | 0.0172 | Yes |
| 2 | MKI67 |  |  | 4 | 0.926 | 0.0362 | Yes |
| 3 | KIF11 |  |  | 6 | 0.870 | 0.0529 | Yes |
| 4 | UBE2C |  |  | 7 | 0.793 | 0.0692 | Yes |
| 5 | KIF20B |  |  | 10 | 0.749 | 0.0823 | Yes |
| 6 | CEP55 |  |  | 11 | 0.747 | 0.0976 | Yes |
| 7 | BUB3 |  |  | 12 | 0.740 | 0.1128 | Yes |
| 8 | KPNA2 |  |  | 20 | 0.667 | 0.1185 | Yes |
| 9 | HIST1H4C |  |  | 21 | 0.659 | 0.1320 | Yes |
| 10 | ASPM |  |  | 27 | 0.639 | 0.1394 | Yes |
| 11 | PCLAF |  |  | 28 | 0.637 | 0.1525 | Yes |
| 12 | DNAJC9 |  |  | 34 | 0.615 | 0.1593 | Yes |
| 13 | CCNB1 |  |  | 37 | 0.599 | 0.1693 | Yes |
| 14 | CKS2 |  |  | 40 | 0.587 | 0.1791 | Yes |
| 15 | HMMR |  |  | 43 | 0.559 | 0.1883 | Yes |
| 16 | NCAPG |  |  | 44 | 0.556 | 0.1997 | Yes |
| 17 | HJURP |  |  | 47 | 0.539 | 0.2085 | Yes |
| 18 | CKS1B |  |  | 50 | 0.530 | 0.2171 | Yes |
| 19 | DBF4 |  |  | 55 | 0.514 | 0.2230 | Yes |
| 20 | KIF14 |  |  | 59 | 0.503 | 0.2299 | Yes |
| 21 | MASTL |  |  | 60 | 0.499 | 0.2401 | Yes |
| 22 | CENPE |  |  | 62 | 0.496 | 0.2492 | Yes |
| 23 | MIS18BP1 |  |  | 64 | 0.495 | 0.2582 | Yes |
| 24 | CCNA2 |  |  | 65 | 0.495 | 0.2683 | Yes |
| 25 | TPX2 |  |  | 66 | 0.488 | 0.2783 | Yes |
| 26 | MYBL2 |  |  | 69 | 0.486 | 0.2860 | Yes |
| 27 | SGO2 |  |  | 73 | 0.478 | 0.2924 | Yes |
| 28 | DLGAP5 |  |  | 76 | 0.475 | 0.2998 | Yes |
| 29 | AURKA |  |  | 78 | 0.474 | 0.3084 | Yes |
| 30 | CDC20 |  |  | 80 | 0.472 | 0.3170 | Yes |
| 31 | UBE2T |  |  | 83 | 0.470 | 0.3243 | Yes |
| 32 | PLK1 |  |  | 89 | 0.464 | 0.3281 | Yes |
| 33 | TUBB4B |  |  | 93 | 0.462 | 0.3341 | Yes |
| 34 | CENPF |  |  | 94 | 0.461 | 0.3436 | Yes |
| 35 | TOP2A |  |  | 95 | 0.457 | 0.3530 | Yes |
| 36 | ZWINT |  |  | 106 | 0.447 | 0.3506 | Yes |
| 37 | AURKB |  |  | 108 | 0.446 | 0.3587 | Yes |
| 38 | CDCA8 |  |  | 115 | 0.437 | 0.3607 | Yes |
| 39 | KIF23 |  |  | 116 | 0.436 | 0.3697 | Yes |
| 40 | DEPDC1 |  |  | 119 | 0.432 | 0.3762 | Yes |
| 41 | MELK |  |  | 123 | 0.427 | 0.3816 | Yes |
| 42 | JPT1 |  |  | 125 | 0.426 | 0.3892 | Yes |
| 43 | SGO1 |  |  | 126 | 0.425 | 0.3979 | Yes |
| 44 | BUB1B |  |  | 129 | 0.421 | 0.4042 | Yes |
| 45 | KNL1 |  |  | 133 | 0.418 | 0.4094 | Yes |
| 46 | RTKN2 |  |  | 141 | 0.413 | 0.4098 | Yes |
| 47 | PIMREG |  |  | 143 | 0.413 | 0.4171 | Yes |
| 48 | GTSE1 |  |  | 146 | 0.411 | 0.4233 | Yes |
| 49 | CCNB2 |  |  | 147 | 0.411 | 0.4317 | Yes |
| 50 | ANLN |  |  | 152 | 0.407 | 0.4355 | Yes |
| 51 | MAD2L1 |  |  | 153 | 0.406 | 0.4438 | Yes |
| 52 | KIFC1 |  |  | 160 | 0.402 | 0.4451 | Yes |
| 53 | ARHGAP11A |  |  | 162 | 0.399 | 0.4522 | Yes |
| 54 | KIF2C |  |  | 165 | 0.397 | 0.4580 | Yes |
| 55 | NDC80 |  |  | 173 | 0.393 | 0.4581 | Yes |
| 56 | FAM83D |  |  | 180 | 0.389 | 0.4592 | Yes |
| 57 | CKLF |  |  | 183 | 0.388 | 0.4648 | Yes |
| 58 | HNRNPH3 |  |  | 185 | 0.387 | 0.4716 | Yes |
| 59 | BUB1 |  |  | 188 | 0.384 | 0.4772 | Yes |
| 60 | KIF22 |  |  | 197 | 0.377 | 0.4757 | Yes |
| 61 | CDCA3 |  |  | 203 | 0.372 | 0.4776 | Yes |
| 62 | TRIP13 |  |  | 209 | 0.370 | 0.4795 | Yes |
| 63 | SMC4 |  |  | 217 | 0.367 | 0.4790 | Yes |
| 64 | SPC25 |  |  | 226 | 0.363 | 0.4772 | Yes |
| 65 | NCAPH |  |  | 232 | 0.359 | 0.4788 | Yes |
| 66 | CENPA |  |  | 243 | 0.352 | 0.4746 | Yes |
| 67 | FEN1 |  |  | 259 | 0.343 | 0.4643 | Yes |
| 68 | TTK |  |  | 262 | 0.340 | 0.4690 | Yes |
| 69 | KNSTRN |  |  | 263 | 0.340 | 0.4760 | Yes |
| 70 | CLSPN |  |  | 274 | 0.336 | 0.4714 | Yes |
| 71 | ESCO2 |  |  | 282 | 0.333 | 0.4702 | Yes |
| 72 | ORC6 |  |  | 283 | 0.333 | 0.4770 | Yes |
| 73 | CACYBP |  |  | 288 | 0.330 | 0.4792 | Yes |
| 74 | C21orf58 |  |  | 294 | 0.328 | 0.4802 | Yes |
| 75 | G2E3 |  |  | 302 | 0.325 | 0.4788 | Yes |
| 76 | MND1 |  |  | 304 | 0.324 | 0.4843 | Yes |
| 77 | CENPN |  |  | 310 | 0.323 | 0.4852 | Yes |
| 78 | FANCI |  |  | 319 | 0.319 | 0.4825 | Yes |
| 79 | TUBA1C |  |  | 322 | 0.318 | 0.4867 | Yes |
| 80 | TEDC1 |  |  | 324 | 0.317 | 0.4921 | Yes |
| 81 | RFC3 |  |  | 337 | 0.314 | 0.4847 | Yes |
| 82 | CDCA5 |  |  | 341 | 0.312 | 0.4877 | Yes |
| 83 | PRC1 |  |  | 350 | 0.310 | 0.4849 | Yes |
| 84 | ANP32B |  |  | 357 | 0.307 | 0.4842 | Yes |
| 85 | ECT2 |  |  | 365 | 0.305 | 0.4825 | Yes |
| 86 | CDCA2 |  |  | 369 | 0.304 | 0.4852 | Yes |
| 87 | BRCA1 |  |  | 373 | 0.302 | 0.4880 | Yes |
| 88 | CKAP2 |  |  | 379 | 0.301 | 0.4884 | Yes |
| 89 | RPA3 |  |  | 381 | 0.300 | 0.4934 | Yes |
| 90 | RRM2 |  |  | 383 | 0.300 | 0.4984 | Yes |
| 91 | CENPW |  |  | 386 | 0.299 | 0.5023 | Yes |
| 92 | PHF19 |  |  | 393 | 0.297 | 0.5015 | No |
| 93 | RACGAP1 |  |  | 399 | 0.293 | 0.5017 | No |
| 94 | HMGB2 |  |  | 424 | 0.288 | 0.4800 | No |
| 95 | POP7 |  |  | 427 | 0.287 | 0.4836 | No |
| 96 | NUSAP1 |  |  | 430 | 0.286 | 0.4872 | No |
| 97 | NUF2 |  |  | 434 | 0.285 | 0.4896 | No |
| 98 | NCAPD2 |  |  | 440 | 0.283 | 0.4897 | No |
| 99 | LMNB1 |  |  | 442 | 0.282 | 0.4943 | No |
| 100 | KIF20A |  |  | 447 | 0.281 | 0.4955 | No |
| 101 | ANP32E |  |  | 453 | 0.280 | 0.4955 | No |
| 102 | MNS1 |  |  | 464 | 0.278 | 0.4897 | No |
| 103 | ATAD2 |  |  | 470 | 0.275 | 0.4896 | No |
| 104 | EZH2 |  |  | 471 | 0.275 | 0.4952 | No |
| 105 | MT2A |  |  | 473 | 0.275 | 0.4997 | No |
| 106 | SHCBP1 |  |  | 477 | 0.274 | 0.5019 | No |
| 107 | PGP |  |  | 503 | 0.270 | 0.4787 | No |
| 108 | KIF4A |  |  | 504 | 0.269 | 0.4842 | No |
| 109 | UBE2S |  |  | 507 | 0.269 | 0.4874 | No |
| 110 | CIP2A |  |  | 522 | 0.266 | 0.4768 | No |
| 111 | SKA3 |  |  | 531 | 0.264 | 0.4730 | No |
| 112 | ALYREF |  |  | 535 | 0.264 | 0.4750 | No |
| 113 | KIF15 |  |  | 538 | 0.263 | 0.4781 | No |
| 114 | CDKN3 |  |  | 539 | 0.263 | 0.4834 | No |
| 115 | SPDL1 |  |  | 560 | 0.258 | 0.4657 | No |
| 116 | DDX39A |  |  | 562 | 0.258 | 0.4699 | No |
| 117 | TMPO |  |  | 584 | 0.253 | 0.4509 | No |
| 118 | SYPL1 |  |  | 608 | -0.255 | 0.4297 | No |
| 119 | FAM111A |  |  | 618 | -0.259 | 0.4246 | No |
Table: GSEA details [plain text format]

  

Fig 2: DOROSZKO\_U3013MG\_CLUSTER\_2: Random ES distribution      
 Gene set null distribution of ES for **DOROSZKO\_U3013MG\_CLUSTER\_2**
